# Augmenter of Liver Regeneration (ALR) Protects Kidney from Ischemia/Reperfusion (I/R) Injury via Regulation of TLR4/MAPK Signaling Pathway

**DOI:** 10.1155/2022/6869730

**Published:** 2022-08-09

**Authors:** Ying Li, Yanying Xiong, Huihui Li, Yan Luo, Ling Zhang, Qin Zhang, Weijian Xiong, Haitao Tu

**Affiliations:** ^1^Department of Nephrology, Chongqing Hospital of Traditional Chinese Medicine, Chongqing 400021, China; ^2^Department of Nephrology, The Second Affiliated Hospital of Chongqing Medical University, Chongqing 400000, China; ^3^Department of Nephrology, The First Affiliated Hospital of Guangzhou University of Chinese Medicine, Guangzhou 510000, China

## Abstract

Toll-like receptor 4 (TLR4) can mediate innate activation and inflammation, and it is typically expressed within the ischemic kidney. Augmenter of liver regeneration (ALR) acts as an immunoregulator with a high expression in the kidney induced by renal ischemia/reperfusion (I/R) injury. Exogenous ALR has indicated a role in protecting the kidney from I/R injury. The protective effect of ALR is due to the immune regulatory function which remains to be elucidated. In this study, rats induced by renal I/R were treated with recombinant human ALR (rhALR) and demonstrated that the animals were protected from kidney I/R injury, implying that the rhALR-treated rats had less tubular damage than those untreated rats. Meanwhile, tubular epithelial cell apoptosis, neutrophil (24 h) and macrophage (72 h) infiltration to tubulointerstitium, and levels of inflammatory cytokines were decreased considerably in the rhALR-treated rats as compared to control. Additionally, rhALR could downregulate mRNA expression of TLR4 endogenous ligands and restrain its activation in renal I/R injury rats. It has also been proved that anti-rhALR antibody blocked the inhibition of rhALR of the immune inflammatory response in hypoxia/reoxygenation (H/R) injury *in vitro*. In rhALR+anti-rhALR antibody-intervened H/R cells, the expression of inflammatory cytokines was upregulated compared with the rhALR-treated cells. Taken together, rhALR could regulate the TLR4 signaling pathway to relieve inflammatory response, thereby protecting renal I/R injury, indicating that ALR is likely to be introduced to develop novel immune therapies for renal I/R injury.

## 1. Introduction

Acute kidney injury (AKI) is recognized as a typical emergency and a high-risk factor endangering the morbidity and mortality of hospitalized patients; strategies on how to impede its progression have caught numerous researchers' attention [1]. Pathogenesis of AKI is yet to be fully understood, and renal ischemic/reperfusion (I/R) injury is broadly known as an independent risk factor for its progression. Apparently, a major factor in the pathogenesis of renal I/R injury is believed to be a strong inflammatory response mediated by the innate immune system of the kidney after ischemia [2, 3]. Numerous studies have revealed that Toll-like receptors are of vital significance in innate immunity by identifying injured renal I/R endogenous ligands [3]. TLR4 is highly expressed in renal tubular epithelial cells (TECs) and immune cells and triggers an innate immune response in early renal I/R by recognizing endogenous ligand-activated mitogen-activated protein kinases (MAPKs) and nuclear factor-*κ*B (NF-*κ*B) signaling pathways [4–6].

Augmenter of liver regeneration (ALR) was extracted and purified from newborn rat livers by Hagiya et al. in 1994 [7]. ALR is a very heat-stable, nonspecific prohepatocyte regenerative factor produced by activated hepatocytes, which specifically promotes liver regeneration and is a new member of a family of conserved functional genes [8, 9]. It has been found that ALR is widely expressed in various tissues and organs in the body and is associated with most disease regression. Besides promoting liver regeneration, ALR can improve mitochondrial function [10] and antifibrosis [11] and modulate immune function [12]. Moreover, ALR is highly expressed in I/R kidneys as a hepatocyte growth factor [13, 14]. Our previous work has indicated that exogenous ALR can protect kidney tissues from I/R injury through inhibiting apoptosis of renal TEC [15–17]. Recent researches have revealed that ALR can also modulate T helper 1 (Th1) cell cytokines and immune cells [18–20].

Our study recently revealed that exogenous ALR alleviates inflammatory responses in a way of inhibiting the TLR4/NF-*κ*B signaling pathway in renal hypoxia-reoxygenation injury *in vitro*, suggesting the protector role of exogenous ALR in renal I/R injury [15, 21]. We evaluated the role of exogenous rhALR in renal I/R injury through the internal and external models of renal I/R injury and found the correlation between the immunomodulatory mechanism of ALR and TLR4 signaling pathway.

## 2. Materials and Methods

### 2.1. Laboratory Animals

Male Sprague-Dawley rats at approximately 200 to 250 g were collected from the Animal Center of Chongqing Medical University. The rats were exposed to a condition of a 12 h light/dark cycle and supplied with regular diets and drinking water ad libitum. Experiments were performed as per guidelines for the treatment and use of laboratory animals of Chongqing Medical University.

### 2.2. Experimental Design

The rats were divided randomly into four of the following groups: sham, I/R, I/R+rhALR1, and I/R+rhALR2. Following ischemia treatment and 12, 24, 36, 48, and 60 h after reperfusion, all animals were intraperitoneally administrated with 400 *μ*g/kg rhALR1, 800 *μ*g/kg rhALR2, or normal saline. The dose of rhALR was chosen with reference to our preliminary experiments. With regard to body weight, identical volume of agent and rhALR were injected at corresponding time points. The experimental animals were put to death at 24, 48, and 72 h after reperfusion (*n* = 8), respectively.

HK2 cells (CRL-2190, American Type Culture Collection, USA) were treated and classified randomly as follows: control, H/R, H/R+rhALR, and H/R+rhALR+anti-rhALR antibody groups. After adding 5% fetal bovine serum, HK2 cells were cultured with Dulbecco-modified Eagle medium at 37°C and 5% CO_2_. 50 *μ*g/mL rhALR and an equal quantity of rhALR+anti-rhALR antibody (Institute for Viral Hepatitis, Chongqing Medical University, China) were added to the H/R+rhALR and H/R+rhALR+anti-rhALR antibody groups, respectively. Finally, the cells were harvested and analyzed twelve hours following reoxygenation but prior to hypoxia for 30 min.

### 2.3. Induction of Kidney I/R Injury

AKI rats were initially anesthetized using sodium pentobarbital (50 mg/kg as per body weight) intraperitoneally, and an incision was made at the midline to expose the abdominal cavity. The renal pedicles of both laterals were occluded using microvascular clamps and lasted for 60 min before withdrawal; the kidneys were then inspected 5 min later. Change in color revealed the occurrence of blood reperfusion [22]. By visual judgement, the rats with incomplete reperfusion were excluded. Subsequently, 1 mL normal saline (100 g) of body weight was administrated by injection into the abdomen at 37°C for fluid compensation during surgery. After occlusion of the renal pedicles, the midline incision was sutured.

### 2.4. HK2 Cell Hypoxia-Reoxygenation Model

The H/R, H/R+rhALR, and H/R+rhALR+anti-rhALR antibody groups were saturated in glucose-free/serum-free medium for 6 h with 1% O_2_, 5% CO_2_, and 94% N_2_ and subsequently kept in a resumed normal oxygenation condition. Cells of the control were cultivated in a normal oxygenation condition.

### 2.5. Assessment of Kidney Function

Blood samples were subsequently obtained using intracardiac puncture and followed by centrifugation at 3000 rpm for 15 min for serum isolation. The renal biochemical parameters were subsequently determined. Meanwhile, blood urea nitrogen and serum creatine were measured using an automated system of Hitachi 747 Analyzer.

### 2.6. Histological Examination

The histological analysis was performed in the following procedures. First, the harvested kidneys of the animals were treated immediately using a 10% formalin solution. The treated tissue fragments were dehydrated with different concentrations of ethanol, embedded in paraffin, prepared into 4 *μ*m thickness sections, and then stained with hematoxylin and eosin solution. Semiquantitative evaluation was also conducted to identify histological changes in tissue damage. Simultaneously, sections of 20 × 400-field were randomly selected to inspect tubular injury of the outer medulla and assigned to five categories as follows: (i) normal; (ii) regions with swollen tubular epithelial cells, degeneration, and necrosis of vacuoles as well as desquamation less than 25% of the tubular profile; (iii) the same alteration between 25% and 50% of the tubular profile; (iv) the same alteration between 50% and 75% of the tubular profile; and (v) the same alteration more than ≥75% of the tubular profile. To avoid some deviations caused by different observers, morphometric tests were conducted by two independent pathologists in a blinded fashion, and the ultimate average scores were calculated with the corresponding parts of the histological injury scores.

### 2.7. Immunohistochemistry Staining

The tissue sections with a thickness of 4 *μ*m were fixed in formalin solution, dewaxed, and boiled in 10 nM sodium citrate buffer (pH = 6.0) for 10 min. Neutrophils were detected. The 7 *μ*m frozen sections were fixed periodate lysine paraformaldehyde for macrophage detection and subsequently blocked with a biotin blocker system. Following additional blocking in a 10 percent natural horse serum, primary antibodies were supplemented to the sections for 60 min, either CD68 or CD11b. As an isotype negative monitor, rat IgG at a corresponding concentration was used. After being treated with methanol and 3% H_2_O_2_ for 5 min, the sections were incubated using biotinylated secondary antibody anti-rabbit IgG. A vector stain ABC kit was applied to the tissues then followed by the 3,3′-diaminobenzidine substrate chromogen solution. Harris' hematoxylin was applied for counterstaining. Cellular infiltration was blindly analyzed; ten consecutive high-power fields were selected at the lateral medulla and corticomedullary junction of each section for assessment. An ocular grid was adopted, and cell count of positive staining for each antibody was calculated and presented in every 10 HPF cells.

### 2.8. Analysis of Apoptosis

TUNEL staining was performed according to the instruction of the apoptosis detection kit (C1091, Beyotime). First, paraffin-embedded sections were dewaxed with xylene for 5 min, then treated with protease K (1.0 *μ*g/mL) at 37°C for 15 min, and treated with 2% H_2_O_2_ at 37°C for 15 min. TdT buffer solution (Beyotime) was added for incubation at room temperature for 30 min, and 1.0 nmol/L digoxin/DUTP was added for incubation at 37°C for 1 h. TUNEL-positive cells were analyzed with ImageJ software (Rawak Software, Inc., Germany).

### 2.9. RNA Extraction and cDNA Synthesis

Complete RNA was extracted from renal tissue and HK2 cells by TRIzol, and cDNA was synthesized by Oligo (dT) 16 and reverse transcriptase kit SuperScript III.

### 2.10. Real-Time PCR Analysis

TaqMan primers and probes specific for TLR4, IL-1*β*, IL-6, TNF-*α*, MCP-1 and MIP-2, HMGB-1, Biglycan, HAS1, HAS2, and HAS3 were obtained from Applied Biosystems, including those for TLR4 (forward, 5′-GCCCAGTGAGAACAGAAAGG-3′, reverse, 5′-AAGGGAAAGGAAGGAAACAT-3′), TNF-*α* (forward, 5′-AAGCCTGTAGCCCACGTCGTA-3′, reverse, 5′-GGCACCACTAGTTGGTTGTCTTTG-3′), IL-1*β* (forward, 5′-CTCTGACAGGCAACCACTTAC-3′, reverse, 5′-GTCCAAATTCAATTCATCCC-3′), IL-6 (forward, 5′-TTGCCTTCTTGGGACTGATG-3′, reverse, 5′-ACTGGTCTGTTGTGGGTGGT-3′), MCP-1 (forward, 5′-TTGCCTTCTTGGGACTGATG-3′, reverse, 5′-ACTGGTCTGTTGTGGGTGGT-3′), MIP-2 (forward, 5′-TCAATGCCTGACGACCCTAC-3′, reverse, 5′-TTTGGACGATCCTCTGAACC-3′), HMGB-1 (forward, 5′-CTCTTAAAGTGCCAGTGTTT-3′, reverse, 5′-ATCATCCAGGACTCATGTTC-3′), Biglycan (forward, 5′-TGAACTCCGCAAGGATGACT-3′, reverse, 5′-CTTCCGCAGAGGGCTAAAGG-3′), HAS1 (forward, 5′-CGCCCTCCTCTTTCCTTCGT-3′, reverse, 5′-CACCGCTTCATAGGTCATCCAC-3′), HAS2 (forward, 5′-GGAATGCTAGTCTTCGGTAA-3′, reverse, 5′-GTCCCAATCACAAAGATAAAGT-3′), HAS3 (forward, 5′-TCTTACTTTCGGGAGTGGCT-3′, reverse, 5′-TATGACTGTGGCGATGAGGA-3′), and *β*-actin (forward, 5′-CCGTAAAGACCTCTATGCCAACA-3′, reverse, 5′-GCTAGGAGCCAGGGCAGTAATC-3′), which were designed using Primer Express Software (Applied Biosystems). As per the manufacturer's instructions of use, cDNA was amplified by 1x Universal Master Mix with the corresponding previously described primers and probes. The PCR conditions were referred to the following: 94°C for 30 s, 55°C for 30 s, and 72°C for 30 s, in a total of 35 cycles. Data analysis was performed based on Sequence Detector V1.9 Analysis Program. The gene expression was normalized against the *β*-actin mRNA expression.

### 2.11. ELISA

IL-1*β*, IL-6, and TNF-*α* were tested using specific ELISA kits as per the manufacturer's protocol. Supernatant or control solution was temporarily applied to 96-well plates added with antibodies and subsequently cultured for 2 h at room temperature. After five cycles of washing, each well was supplemented with detecting antibodies, incubated for 2 h at room temperature, and provided with 100 *μ*L of substrate solution. An addition of 100 *μ*L terminator was applied to the wells and incubated for 90 min. The absorbance at 450 nm was detected using an ELISA reader.

### 2.12. Statistical Analysis

Data information was expressed as the mean + standard deviation after statistical analysis by utilizing the SPSS 22.0 software. The minimum significant difference (LSD) was evaluated among certain groups. Pairwise comparison was performed using *T* tests and multiple group comparison using one-way variance analysis (ANOVA). The value of *P* less than 0.05 was considered statistically significant.

## 3. Results

### 3.1. rhALR Protects Kidney against Renal I/R Injury

**Figure 1:**
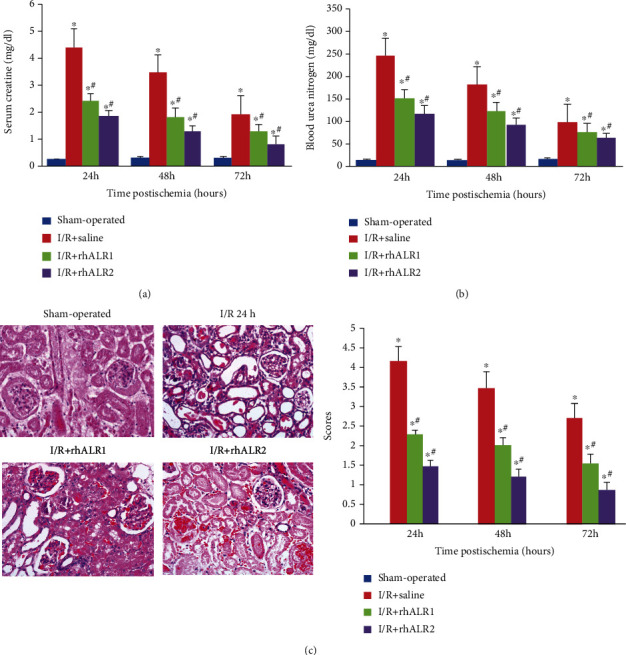
rhALR attenuates renal damage after I/R injury induction. (a, b) After 60 min of bilateral renal ischemia, blood samples were collected at 24, 48, and 72 h, followed by renal reperfusion, and serum creatinine and blood urea nitrogen were measured. The increase of two indexes in the rhALR1 and rhALR2 groups was withdrawn (*P* < 0.05). Urea nitrogen and serum creatinine in the I/R+rhALR2 group were lower than those in the I/R+rhALR1 group (*P* < 0.05). (c) HE staining of 24 h renal slices in the I/R group showed severe renal tubule necrosis. Renal morphology was preserved after rhALR1 and rhALR2 treatment (magnification, ×200). Histological changes were assessed semiquantitatively for tubular necrosis. The tubular damage score was given by two uninformed observers. Data were expressed as the mean ± SD. ^∗^*P* < 0.05 as compared to the sham group, and ^#^*P* < 0.05 as compared to the I/R+saline group.

In comparison with the sham group, rats induced by renal I/R injury had an increase in levels of blood urea nitrogen and serum creatine. The highest level at 24 h and the lowest at 72 h after surgery showed that renal failure had peaked at 24 h and had started to heal afterwards. Blood urea nitrogen and serum creatine levels in the rhALR1 or rhALR2 group were markedly decreased compared to the saline-treated control animals at 24 h and 72 h after surgery, respectively, while those in the I/R+rhALR2 group were lower than those in the I/R+rhALR1 group. All the values of *P* were less than 0.05 (Figures 1(a) and 1(b)). Compared to the tissues from the sham operation, renal I/R surgery resulted in substantial changes in histological levels in rats. Renal I/R injury induced by surgery caused extensive tubular architecture degeneration and dilatation, necrosis, swelling, luminal obstruction with boundary failure, and polymorphonuclear neutrophil infiltration. At 24 h, the hallmark histological characteristics of ischemic injury were noticeable but relieved after reperfusion at 72 h. In comparison, the tissues of the rhALR1 or rhALR2 group displayed less histological characteristics for renal injury. In vehicle-treated control rats, semiquantitative lesion tests reported significantly higher scores compared to the rats in the rhALR1 and rhALR2 groups following reperfusion injury. Conversely, the scores of the rhALR2 group were lower than those in the rhALR1 group (*P* < 0.05) (Figure 1(c)).

### 3.2. rhALR Reduces Tubular Epithelial Cell Apoptosis in the I/R Kidney

**Figure 2:**
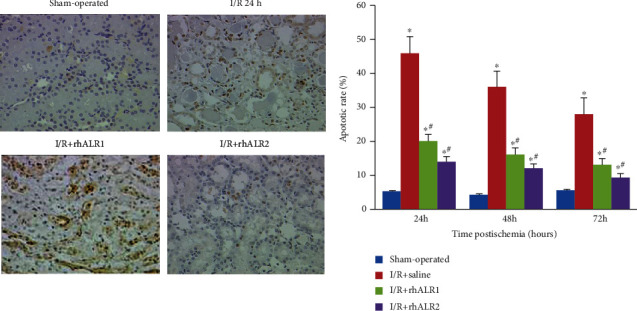
rhALR attenuates renal damage after I/R injury. The 24-hour representative kidney sections were shown above. TUNEL staining showed that TUNEL-positive apoptotic cells in the renal tubules of the sham operation group were few at first, but the number of TUNEL-positive apoptotic cells increased 24 h after operation and reached a peak at 72 h. The renal function level of the I/R group was the same. The number of TUNEL-positive cells in the rhALR1 and rhALR2 groups was lower than that in the I/R group at these three time points (magnification ×400). All data were presented as the mean ± standard deviation. ^∗^*P* < 0.05 vs sham, ^#^*P* < 0.05 vs. I/R+saline.

Quantification of cell apoptosis was performed and verified using TUNEL staining. No apoptotic cells revealed TUNEL positive in sham group rat kidneys either in the outer stripe of the outer medulla or renal cortex. And there was a significant increase in renal I/R, which reached a peak 24 h after reperfusion. When compared with the I/R group, the average number of apoptotic cells in the I/R+rhALR1 and I/R+rhALR2 groups was notably reduced. In the comparison of the I/R+ rhALR1 group, the apoptosis of the I/R+rhALR2 group was decreased at the specified time (*P* < 0.05) (Figure 2).

### 3.3. rhALR Reduces Interstitial Infiltration in the I/R Kidney

**Figure 3:**
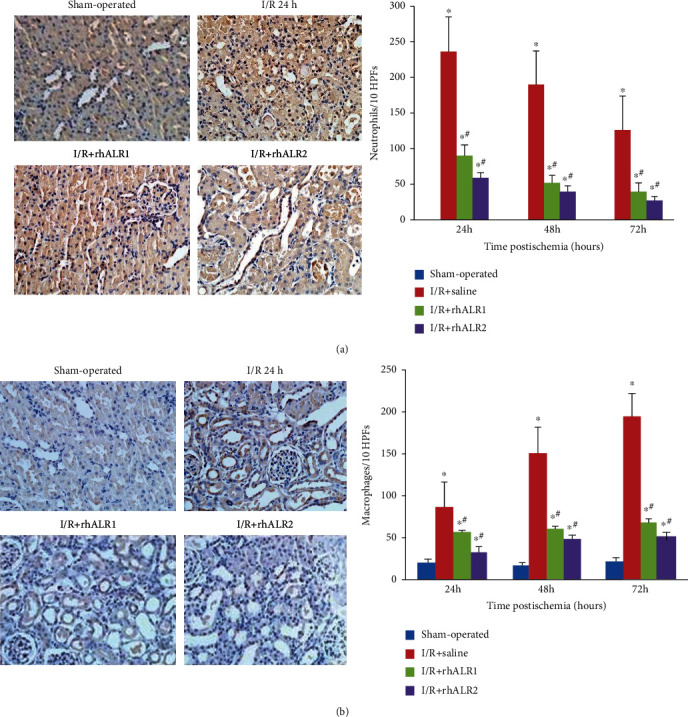
rhALR treatment results in less interstitial inflammatory cell infiltration after renal I/R injury induction. Immunostaining of CD11b (a) and CD68 (b) in the extrarenal medulla of rats was induced by sham operation or I/R injury 24-72 h after surgery. Sections at 72 h are shown in (a, b) (magnification: ×200). 10 HPF cells were counted in each section. After the induction of renal I/R injury, the number of neutrophils and macrophages increased rapidly and reached the peak at 24 and 72 h, respectively. Exogenous rhALR reduced neutrophils and macrophage infiltration at both time points. All data were presented as the mean ± SD. ^∗^*P* < 0.05 vs. sham, ^#^*P* < 0.05 vs. I/R+saline group.

Immunochemistry has established a few neutrophils and macrophages in sham-operated rat kidneys. In renal I/R injury-induced rats, high numbers of neutrophils and macrophages invaded the renal interstitium. After renal I/R injury induction, the number of neutrophils peaked at 24 h, while at 72 h postinduction, macrophages reached the top. The mean number of inflammatory cells was markedly reduced in the rhALR group as compared to saline-treated control (*P* < 0.05) (Figures 3(a) and 3(b)).

### 3.4. rhALR Attenuates the Cytokines and Chemokines within the Kidney after I/R Injury

**Figure 4:**
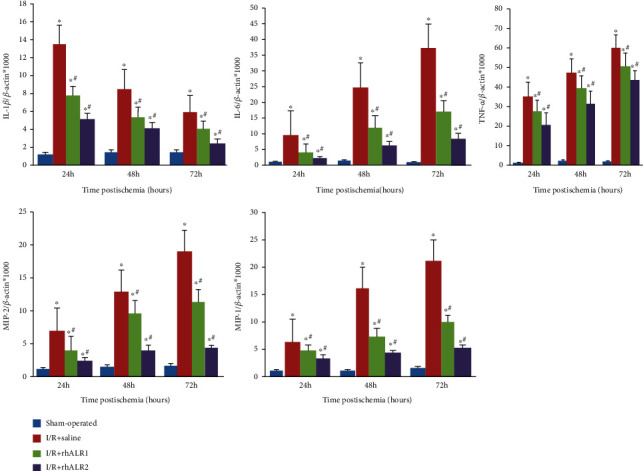
rhALR suppressed renal inflammatory cytokine and chemokine production after I/R injury. The mRNA expressions of inflammatory cytokines and chemokines (IL-1*β*, IL-6, TNF-*α*, MIP-2, and MCP-1) were detected by real-time PCR at 24, 48, and 72 h after renal I/R injury treatment. All data were expressed as the mean ± SD. ^∗^*P* < 0.05 vs. sham, ^#^*P* < 0.05 vs. I/R+saline group.

**Figure 5:**
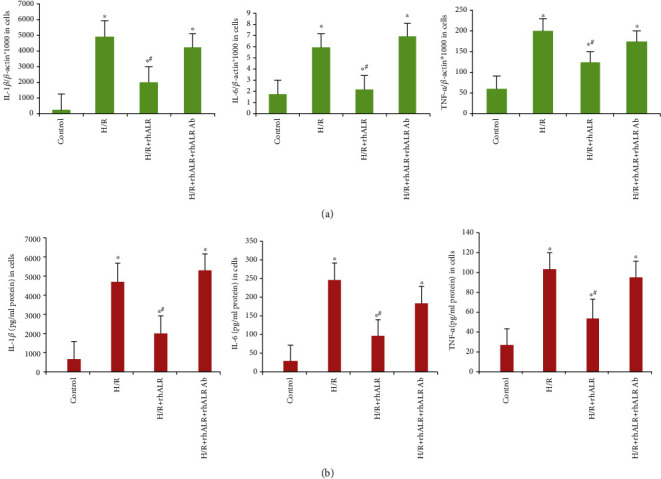
rhALR alleviates inflammation in H/R-induced HK2 cells. (a, b) Real-time PCR and ELISA analysis showed that the mRNA and protein expressions of IL-1*β*, IL-6, and TNF-*α* were upregulated after H/R induced reoxygenation for 12 h in HK2 cells. No above changes were observed in the H/R+rhALR group and the negative control group. And exogenous anti-rhALR Ab evidently blocked the anti-inflammatory effect of rhALR. All data obtained were expressed as the mean ± SD. ^∗^*P* < 0.05 vs. negative control, ^#^*P* < 0.05 vs. H/R.

Increasing research has shown that cytokines and chemokines produced by TLR4 mediated the robust inflammatory response of renal I/R injury. It is therefore that downstream cytokines and chemokines of TLR4 mRNA expression were detected by real-time PCR. IL-1*β*, IL-6, MIP-2, and MCP-1 mRNAs were significantly upregulated in I/R kidney from 24 h to 72 h postinduction, peaking at 24 h (Figure 4). The TNF-*α* mRNA level was significantly increased and peaked at 72 h postoperation (*P* < 0.05). Both rhALR1 and rhALR2 markedly downregulated the cytokine mRNA levels after renal I/R injury induction. The cytokine mRNA expression was decreased in the I/R+ rhALR2 group as compared to the I/R+rhALR1 group (*P* < 0.05). We further measured the mRNA and protein levels of IL-1*β*, IL-6, and TNF-*α* in HK2 cells 12 h after H/R. In rhALR intervened H/R cells, all were upregulated as compared with control (*P* < 0.05), which were increased substantially in the H/R+rhALR+anti-rhALR Ab group as compared to the H/R+rhALR group (*P* < 0.05) (Figure 5). The findings indicated that rhALR could protect I/R-induced kidneys and it was associated with inflammatory cell infiltration inhibition and inflammatory cytokine and chemokine expression in the kidney.

### 3.5. rhALR Downregulates TLR4 and Endogenous Ligand Expression after I/R Injury

**Figure 6:**
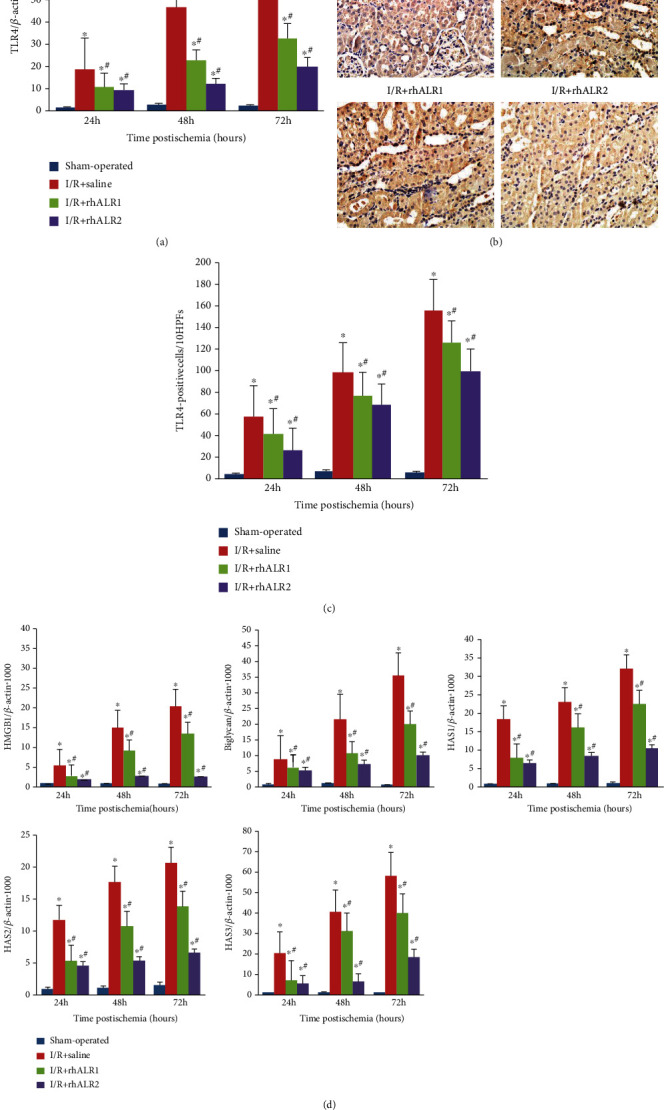
rhALR downregulated the expression of TLR4 and endogenous ligands after I/R injury. (a, b) TLR4 mRNA expression after renal I/R injury was measured by immunohistochemical (magnification, ×200) and real-time PCR methods, respectively. TLR4 expression was increased markedly in kidneys after rat I/R injury. Intraperitoneal injection of rhALR markedly decreased the expression of TLR4 vs. I/R+saline. TLR4 protein expression in untreated control cells was low. (c) The endogenous ligand mRNA expression began to increase since 24 h and reached the peak at 72 h following I/R injury. Expression of these mRNA downregulation in I/R+rhALR1 and I/R+rhALR2 at the same points. All data were shown as the mean ± SD. ^∗^*P* < 0.05 vs. sham, ^#^*P* < 0.05 vs. I/R+saline.

To confirm the effect of rhALR on TLR4 signaling, immunochemistry and real-time PCR were conducted for TLR4 detection. 24 h after renal I/R induction, the levels of TLR4 mRNA and protein in the sham operation group were lower than that of the other three groups. TLR4 expression was lower in kidneys of the rhALR group than those of saline-treated rats in a dosage-dependent manner (*P* < 0.05) (Figure 6(a)). Immunochemistry showed that TLR4 was mainly expressed in renal tubular epithelial cells and infiltrative inflammatory cells after I/R injury (Figure 6(b)). Real-time PCR detection of the TLR4 expression level at each time point revealed that the I/R+rhALR2 group decreased more significantly than the I/R+rhALR group (*P* < 0.05) (Figures 6(c)). Expression of the endogenous ligand mRNA was greatly elevated after renal I/R 24 h and peaked at 72 h. In the rhALR-treated group, the expression level of mRNA was substantially decreased as compared to the saline-treated group (*P* < 0.05) (Figure 6(d)).

This study revealed that TLR4 mRNA expression was consistent with that of its endogenous ligands, suggesting that TLR4 was activated through recognizing endogenous TLR4 ligands released by injured kidney cells. Exogenous ALR probably restrain the activation of TLR4 receptor and its downstream signaling by inhibiting endogenous TLR4 ligand mRNA expression.

## 4. Discussion

In the early stage of renal I/R, oxygen free radicals and lipid peroxidation directly lead to kidney damage which in turn causes TLR4 ligand expression or release endogenously [23, 24]. TLR4 interacts with ligands resulting in MAPK signaling activation via MyD88-independent or TRIF-independent pathways [25]. The activation of transcriptional target gene expression (including cytokines, chemokines, and molecules of adhesion) and upregulation of proinflammatory cytokines and chemokines ultimately contribute to innate immune response [26]. MAPKs, one of the significant signaling pathways in eukaryotes, play a critical role in controlling cytoplasm gene expression and function. Five known signaling pathways involving MAPKs have now been identified. ERK1/2 signaling mainly controls cell growth and differentiation; signaling pathways for JNK and p38 are of vital significance in stress reactions of inflammation and apoptosis [27, 28]. In this research, we found that the endogenous TLR4 ligands (HMGB1, Biglycan, HAS1, HAS2, and HAS3) were markedly increased both *in vivo* and *in vitro* at the mRNA level in the renal I/R injury model, and TLR4 was upregulated simultaneously. MAPKs are activated, cytokines and chemokines are strengthened, and tubulointerstitial tissue is invaded by neutrophils and macrophages, indicating that the signaling pathway of TLR4 poses an overactivation condition throughout renal injury.

ALR is a widely expressed antiapoptotic factor in eukaryotes, which has the ability to promote the proliferation of renal tubular cells and protect cells from injury. Through experiments, we found that ALR expression was significantly increased in the kidney of AKI ischemia rats. Inhibiting renal tubular cell apoptosis and promoting ALR regeneration are a way to protect renal cells from AKI damage by mediating p53 and activating the Akt signaling pathway [29]. Recent studies have highlighted that immune regulation is another unique biological role of ALR. Gupta et al. assumed that a possible mechanism for ALR in liver regeneration is to inhibit certain activities of the organ's inherent natural killer cells [30, 31]. We found that exogenous ALR inhibits the activation of NF-*κ*B by downregulating the expression of TLR4 protein and mRNA, thereby inhibiting the inflammatory response of NRK-52E cells induced through hypoxia and reoxygenation [21]. We hypothesized that rhALR protects renal function by inhibiting the release of endogenous TLR4 ligands and the activation of TLR4 signaling pathways. Therefore, we had the rat renal I/R model for further analysis to verify this hypothesis [32, 33]. Further research found that exogenous rhALR protected the ischemic renal system by attenuating neutrophil and macrophage tubulointerstitial infiltration and downregulating inflammatory cytokines and chemokine levels. In *in vitro* model experiments, we found the anti-inflammatory effect of exogenous rhALR in H/R-induced HK2 cells, which was confirmed by lower cytokine expression (IL-1*β*, IL-6, and TNF-*α*) in rhALR-treated cells than in non-H/R-induced cells. Cytokines, chemokines, and adhesion factors can induce renal cell apoptosis or dysfunction and cause renal damage caused by an increased number of inflammatory cells produced by granzyme and perforin [3, 34]. Although various cytokines and chemokines may continue to be expressed in autocrine and paracrine ways (e.g., IL-1 promotes TNF-alpha and IL-6 production, and TNF-alpha stimulates autocrine continuous cell expression), a number of studies have shown that the development of most proinflammatory factors and chemokines is mediated by MAPKs and NF-*κ*B signaling pathways triggered by TLR4 [27, 35–37]. Therefore, our further study showed that different exogenous rhALR administration concentrations could significantly reduce relevant TLR4 mRNA expression and limit its activation and downstream signal molecules for rats with kidney I/R injury. It suggested that rhALR inhibited the activation of TLR4 signaling, restrained renal inflammation, and ultimately avoided renal injury progression.

The study indicated that ERK phosphorylation increased in ischemia kidney, but the exogenous rhALR could markedly downregulate the ERK phosphorylation. In fact, ERK signaling is the key way to control cell growth, proliferation, and inflammatory genes. Our previous studies have confirmed that in a variety of study models, ALR promotes TEC proliferation [15, 29, 33, 38]. Several studies have found that the signaling pathway of PI3K/Akt is another significant pathway for cell proliferation, so we speculated that the upregulation of Akt phosphorylation activates the signaling pathway of PI3K/Akt in TECs, leading to TEC proliferation [39–41]. In addition, it has been proposed that TNF-alpha is a proapoptotic factor that can induce apoptosis. This may be another mechanism of the protective effect of ALR on the ischemic kidney [42, 43].

## 5. Conclusion

The present study clarified that rhALR acted as an immunoregulator in renal I/R injury, most likely acting through the suppression of innate immune responses induced by TLR4. In renal I/R injury treatment, these promising findings placed ALR as a candidate for novel molecular therapy.

## Data Availability

The data used to support the findings of this study are included within the article.

## References

[1] Zhang L., Sun D., Bao Y., Shi Y., Cui Y., Guo M. (2017). Nerolidol protects against LPS-induced acute kidney injury via inhibiting TLR4/NF-*κ*B signaling. *Phytotherapy Research*.

[2] Tan X., Zheng X., Huang Z., Lin J., Xie C., Lin Y. (2017). Involvement of S100A8/A9-TLR4-NLRP3 inflammasome pathway in contrast-induced acute kidney injury. *Cellular Physiology and Biochemistry*.

[3] Andrade-Silva M., Cenedeze M. A., Perandini L. A. (2018). TLR2 and TLR4 play opposite role in autophagy associated with cisplatin-induced acute kidney injury. *Clinical Science (London, England)*.

[4] Chen X., Zheng X., Zhang M. (2018). Nuciferine alleviates LPS-induced mastitis in mice via suppressing the TLR4-NF-*κ*B signaling pathway. *Inflammation Research*.

[5] Ma X., Yan L., Zhu Q., Shao F. (2017). Puerarin attenuates cisplatin-induced rat nephrotoxicity: the involvement of TLR4/NF-*κ*B signaling pathway. *PLoS One*.

[6] Mohamed A. F., Safar M. M., Zaki H. F., Sayed H. M. (2017). Telluric acid ameliorates endotoxemic kidney injury in mice: involvement of TLR4, Nrf2, and PI3K/Akt signaling pathways. *Inflammation*.

[7] Hagiya M., Francavilla A., Polimeno L. (1994). Cloning and sequence analysis of the rat augmenter of liver regeneration (ALR) gene: expression of biologically active recombinant ALR and demonstration of tissue distribution. *Proceedings of the National Academy of Sciences*.

[8] Chen X., Li Y., Wei K. (2003). The potentiation role of hepatopoietin on activator protein-1 is dependent on its sulfhydryl oxidase activity. *The Journal of Biological Chemistry*.

[9] Lu C., Li Y., Zhao Y. (2002). Intracrine hepatopoietin potentiates AP-1 activity through JAB1 independent of MAPK pathway. *The FASEB Journal*.

[10] Abe J., Baines C. P., Berk B. C. (2000). Role of mitogen-activated protein kinases in ischemia and reperfusion injury. *Circulation Research*.

[11] Lee J., Hofhaus G., Lisowsky T. (2000). Erv1p from Saccharomyces cerevisiae is a FAD-linked sulfhydryl oxidase. *FEBS Letters*.

[12] Li Q., Liu D. W., Zhang L. M., Zhu B., He Y. T., Xiao Y. H. (2005). Effects of augmentation of liver regeneration recombinant plasmid on rat hepatic fibrosis. *World Journal of Gastroenterology: WJG*.

[13] Pu T., Liao X. H., Sun H. (2017). Augmenter of liver regeneration regulates autophagy in renal ischemia-reperfusion injury via the AMPK/mTOR pathway. *Apoptosis*.

[14] Ibrahim S., Weiss T. S. (2019). Augmenter of liver regeneration: essential for growth and beyond. *Cytokine & Growth Factor Reviews*.

[15] Li Y., Zhang L., Liu Q., Chen G. T., Sun H. (2014). Exogenous augmenter of liver regeneration (ALR) attenuates inflammatory response in renal hypoxia re-oxygenation injury. *Renal Failure*.

[16] Chen G. T., Zhang L., Liao X. H. (2014). Augmenter of liver regeneration ameliorates renal fibrosis in rats with obstructive nephropathy. *Bioscience Reports*.

[17] Huang L. L., Liao X. H., Sun H., Jiang X., Liu Q., Zhang L. (2019). Augmenter of liver regeneration protects the kidney from ischaemia-reperfusion injury in ferroptosis. *Journal of Cellular and Molecular Medicine*.

[18] Ibrahim S., Dayoub R., Melter M., Weiss T. S. (2018). Bile acids down-regulate the expression of augmenter of liver regeneration (ALR) via SHP/HNF4*α*1 and independent of Egr-1. *Experimental and Molecular Pathology*.

[19] Kumar S., Wang J., Rani R., Gandhi C. R. (2016). Hepatic deficiency of augmenter of liver regeneration exacerbates alcohol-induced liver injury and promotes fibrosis in mice. *PLoS One*.

[20] Vodovotz Y., Prelich J., Lagoa C. (2013). Augmenter of liver regeneration (ALR) is a novel biomarker of hepatocellular stress/inflammation: in vitro, in vivo and in silico studies. *Molecular Medicine*.

[21] Yan R., Li Y., Zhang L. (2015). Augmenter of liver regeneration attenuates inflammation of renal ischemia/reperfusion injury through the NF-kappa B pathway in rats. *International Urology and Nephrology*.

[22] Ulu N. (2009). *Vascular Function in Chronic End-Organ Damage: Pharmacological Characterization of Vasomotor Function in Systemic Vasculature. Chapter 8: Effects of Chronic EGFR Inhibition on Vascular Contractile Function in a Rat Model of Kidney I/R Injury*.

[23] Goligorsky M. S. (2011). TLR4 and HMGB1: partners in crime?. *Kidney International*.

[24] Nath K. A., Belcher J. D., Nath M. C. (2018). Role of TLR4 signaling in the nephrotoxicity of heme and heme proteins. *American Journal of Physiology-Renal Physiology*.

[25] Zmonarski S. C., Banasik M., Madziarska K., Mazanowska O., Krajewska M. (2019). The role of toll-like receptors in multifactorial mechanisms of early and late renal allotransplant injury, with a focus on the TLR4 receptor and mononuclear cells. *Advances in Clinical and Experimental Medicine: Official Organ Wroclaw Medical University*.

[26] O'Neill S., Humphries D., Tse G. (2015). Heat shock protein 90 inhibition abrogates TLR4-mediated NF-*κ*B activity and reduces renal ischemia-reperfusion injury. *Scientific Reports*.

[27] Qi M., Zheng L., Qi Y. (2015). Dioscin attenuates renal ischemia/reperfusion injury by inhibiting the TLR4/MyD88 signaling pathway via up-regulation of HSP70. *Pharmacological Research*.

[28] Yan R., Zhang L., Xia N., Liu Q., Sun H., Guo H. (2015). Knockdown of augmenter of liver regeneration in HK-2 cells inhibits inflammation response via the mitogen-activated protein kinase signaling pathway. *Inflammation Research*.

[29] Liao X. H., Zhang L., Chen G. T. (2014). Augmenter of liver regeneration inhibits TGF-*β*1-induced renal tubular epithelial-to-mesenchymal transition via suppressing T*β*R II expression in vitro. *Experimental Cell Research*.

[30] Gupta P., Sata T. N., Ahamad N. (2019). Augmenter of liver regeneration enhances cell proliferation through the microRNA‐26a/Akt/cyclin D1 pathway in hepatic cells. *Hepatology Research*.

[31] Gupta P., Sata T. N., Yadav A. K. (2019). TGF-*β* induces liver fibrosis via miRNA-181a-mediated down regulation of augmenter of liver regeneration in hepatic stellate cells. *PLoS One*.

[32] Huang L. L., Long R. T., Jiang G. P. (2018). Augmenter of liver regeneration promotes mitochondrial biogenesis in renal ischemia-reperfusion injury. *Apoptosis*.

[33] Jiang X., Liao X. H., Huang L. L., Sun H., Liu Q., Zhang L. (2019). Overexpression of augmenter of liver regeneration (ALR) mitigates the effect of H_2_O_2_-induced endoplasmic reticulum stress in renal tubule epithelial cells. *Apoptosis*.

[34] Niu X., Yao Q., Li W. (2019). Harmine mitigates LPS-induced acute kidney injury through inhibition of the TLR4-NF-*κ*B/NLRP3 inflammasome signalling pathway in mice. *European Journal of Pharmacology*.

[35] Tan J., He J., Qin W., Zhao L. (2019). Quercetin alleviates lipopolysaccharide-induced acute kidney injury in mice by suppressing TLR4/NF-*κ*B pathway. *Nan Fang Yi Ke Da Xue Xue Bao= Journal of Southern Medical University*.

[36] Liu B., Ding F., Hu D. (2018). Human umbilical cord mesenchymal stem cell conditioned medium attenuates renal fibrosis by reducing inflammation and epithelial-to-mesenchymal transition via the TLR4/NF-*κ*B signaling pathway in vivo and in vitro. *Stem Cell Research & Therapy*.

[37] Michel H. E., Menze E. T. (2019). Tetramethylpyrazine guards against cisplatin-induced nephrotoxicity in rats through inhibiting HMGB1/TLR4/NF-*κ*B and activating Nrf2 and PPAR-*γ* signaling pathways. *European Journal of Pharmacology*.

[38] Xia N., Yan R. Y., Liu Q. (2015). Augmenter of liver regeneration plays a protective role against hydrogen peroxide-induced oxidative stress in renal proximal tubule cells. *Apoptosis*.

[39] Hu C., Li L., Ding P. (2018). Complement inhibitor CRIg/FH ameliorates renal ischemia reperfusion injury via activation of PI3K/AKT signaling. *The Journal of Immunology*.

[40] Hu S., Zhang Y., Zhang M. (2016). Aloperine protects mice against ischemia-reperfusion (IR)-induced renal injury by regulating PI3K/AKT/mTOR signaling and AP-1 activity. *Molecular Medicine*.

[41] Li J., Chen Q., He X. (2018). Dexmedetomidine attenuates lung apoptosis induced by renal ischemia-reperfusion injury through *α* (2) AR/PI3K/Akt pathway. *Journal of Translational Medicine*.

[42] Wang C., Hao Z., Zhou J., Zhang L., Sun Y., Liang C. (2017). Rutaecarpine alleviates renal ischemia reperfusion injury in rats by suppressing the JNK/p38 MAPK signaling pathway and interfering with the oxidative stress response. *Molecular Medicine Reports*.

[43] Yang C. C., Yao C. A., Yang J. C., Chien C. T. (2014). Sialic acid rescues repurified lipopolysaccharide-induced acute renal failure via inhibiting TLR4/PKC/gp91-mediated endoplasmic reticulum stress, apoptosis, autophagy, and pyroptosis signaling. *Toxicological Sciences*.

